# Body Mass Index Changes and Insulin Resistance at Age 4: A Prospective Cohort Study

**DOI:** 10.3389/fendo.2022.872591

**Published:** 2022-05-23

**Authors:** Hye Jin Lee, Youn-Hee Lim, Yun-Chul Hong, Choong Ho Shin, Young Ah Lee

**Affiliations:** ^1^ Department of Pediatrics, Hallym University Kangnam Sacred Heart Hospital, Seoul, South Korea; ^2^ Section of Environmental Health, Department of Public Health, University of Copenhagen, Copenhagen, Denmark; ^3^ Institute of Environmental Medicine, Seoul National University Medical Research Center, Seoul, South Korea; ^4^ Environmental Health Center, Seoul National University College of Medicine, Seoul, South Korea; ^5^ Department of Preventive Medicine, Seoul National University College of Medicine, Seoul, South Korea; ^6^ Department of Pediatrics, Seoul National University Children’s Hospital, Seoul, South Korea

**Keywords:** child, preschool, insulin resistance, body mass index, adiposity rebound

## Abstract

**Objectives:**

The objective of this study is to investigate whether body mass index (BMI) changes are associated with fasting glucose and insulin resistance (IR) in early childhood.

**Methods:**

From the Environment and Development of Children (EDC) cohort, 334 children who visited at ages 2 and 4 were included in this study. Height and weight were measured at ages 2 and 4, and fasting glucose and insulin were assessed at age 4. Homeostatic model assessment of insulin resistance (HOMA-IR) was calculated as insulin (μIU/ml) × glucose (mg/dl)/405. The BMI Z-score [BMI (Z)] quartiles for each age group were defined as Q4, ≥75th percentile; Q2–3, 25th to 75th percentile; and Q1, <25th percentile. Glucose, insulin, and the HOMA-IR were compared between groups according to the change in BMI (Z) from age 2 to 4.

**Results:**

Children who stayed in Q4 at both ages had higher fasting glucose (92.2 vs. 88.0 and 87.1 mg/dl), insulin (3.2 vs. 2.5 and 2.3 μIU/ml), and HOMA-IR (0.68 vs. 0.54 and 0.52) than children who stayed in Q1 or Q2–3 (all P<0.01). Children in Q4 at both ages had higher fasting glucose than children whose BMI (Z) increased from Q1 or Q2–3 to Q4 (92.2 vs. 87.3, P<0.001). The BMI (Z) category at age 2 of children who were in Q2–3 at age 4 did not affect glucose or IR at 4 years.

**Conclusion:**

The group of children within the highest BMI (Z) quartile at both 2 and 4 years of age had higher fasting glucose and IR at age 4 than any other BMI (Z) change group.

## Introduction

Insulin resistance (IR) refers to a decreased tissue response to insulin stimulation, which can lead to failure to maintain glucose homeostasis, type 2 diabetes (T2DM), and metabolic syndrome. Obesity is a well-known risk factor for IR (1). Early-life conditions such as exposure to maternal gestational hyperglycemia (2), low birth weight (3–5), and early adiposity rebound (6–8) increase the risk of developing obesity or T2DM later in life.

Early childhood is a critical period for adiposity change intervention to prevent adulthood obesity (9). The early childhood body mass index (BMI) decreases after the first year of life, reaches a nadir at approximately 6 years of age, and then increases again throughout childhood. The earlier the adiposity rebound, the higher the risk for obesity and IR (7). An above-average BMI in early childhood, even at levels below the international definitions of overweight, is positively associated with T2DM in adult life (10). Since previous studies have focused on the relationship of children’s BMI after adiposity rebound with IR or later T2DM (10–13), the longitudinal relationship of early childhood BMI changes with fasting blood glucose (FBG) levels or IR remains to be determined.

We aimed to investigate the association between the BMI Z-score [BMI (Z)] change and IR in early childhood. In a prospective birth cohort, we analyzed whether changes in BMI (Z) quartile from age 2 to 4 affected FBG and IR at age 4. We hypothesized that children in the highest BMI quartile or whose BMI quartile increased from 2 to 4 years of age would have greater IR at age 4.

## Materials and Methods

### Subjects

The data from the Environment and Development of Children (EDC) cohort were used in this study. The EDC cohort is a prospective community-based birth cohort that is followed to investigate the effect of environmental exposures from the prenatal period to early childhood on physical and neurobehavioral development (14). A total of 726 children, consisting of 2-year-old (n=425) and 4-year-old children (n=301), were initially enrolled. Among the 425 children aged 2 years enrolled in 2012–2013, 343 (80.7%) came to the 4-year-old follow-up. After excluding the children without blood sample (n=2), BMI (n=2), birth weight (n=1), or gestational age (n=4) data, 334 children (179 boys and 155 girls) were finally included in this analysis (**Supplementary Figure S1**).

### Questionnaires

A questionnaire including the birth history (birth weight and gestational age), the duration of exclusive breastfeeding, parental height and weight, and maternal education (graduated from high school, college, or graduate school) were completed by the parents.

### Measurements

At each visit at ages 2 and 4, height and weight were measured using a Harpenden stadiometer (Holtian Ltd, Crymych, United Kingdom) and a digital scale (150 A; Cas Co., Ltd., Seoul, South Korea), respectively. BMI was calculated as weight (kg) divided by height (m) squared. The height, weight, and BMI (Z) were calculated for each subject based on the 2007 Korean National Growth Charts (15). Preterm birth was defined as birth at <37 weeks of gestation. Small for gestational age (SGA) was defined as <10th percentile of weight for gestational age using Fenton growth charts (2013) (16). For the follow-up examination at age 4, blood samples were collected after >8 h of fasting. Homeostatic model assessment of IR (HOMA-IR) was calculated as fasting insulin (μIU/ml) × FBG (mg/dl)/405.

### Statistical Analysis

**Figure 1:**
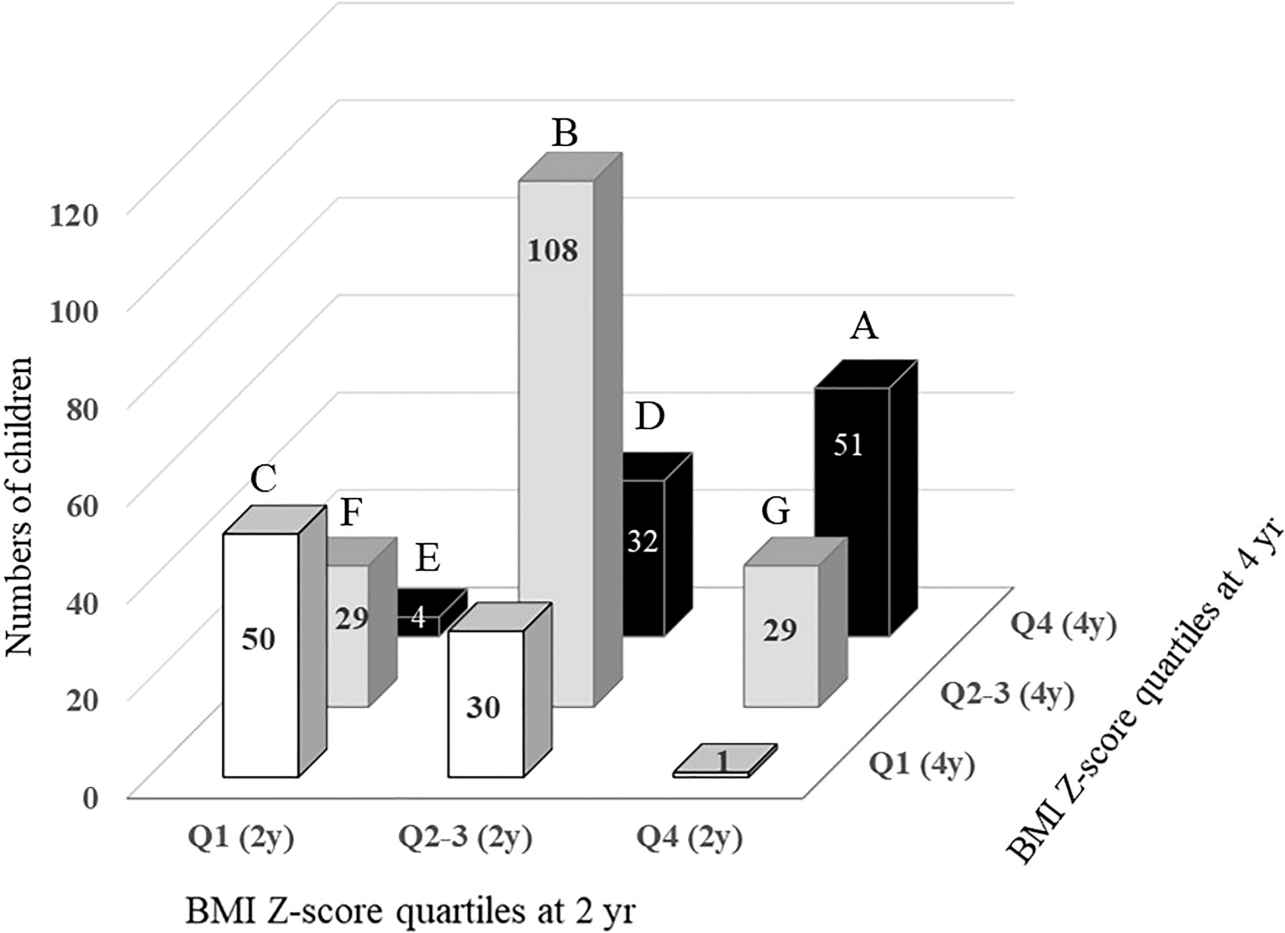
Changes in BMI Z-score quartile in children between the ages of 2 and 4. A, children in Q4 at both ages 2 and 4. B, children in Q2-3 at both ages 2 and 4. C, children in Q1 at both ages 2 and 4. D, children with BMI Z-score quartile increasing from Q2-3 at age 2 to Q4 at age 4. E, children with BMI Z-score quartile increasing from Q1 at age 2 to Q4 at age 4. F, children with BMI Z-score quartile increasing from Q1 at age 2 to Q2-3 at age 4. G, children with BMI Z-score quartile decreasing from Q4 at age 2 to Q2-3 at age 4.

**Figure 2:**
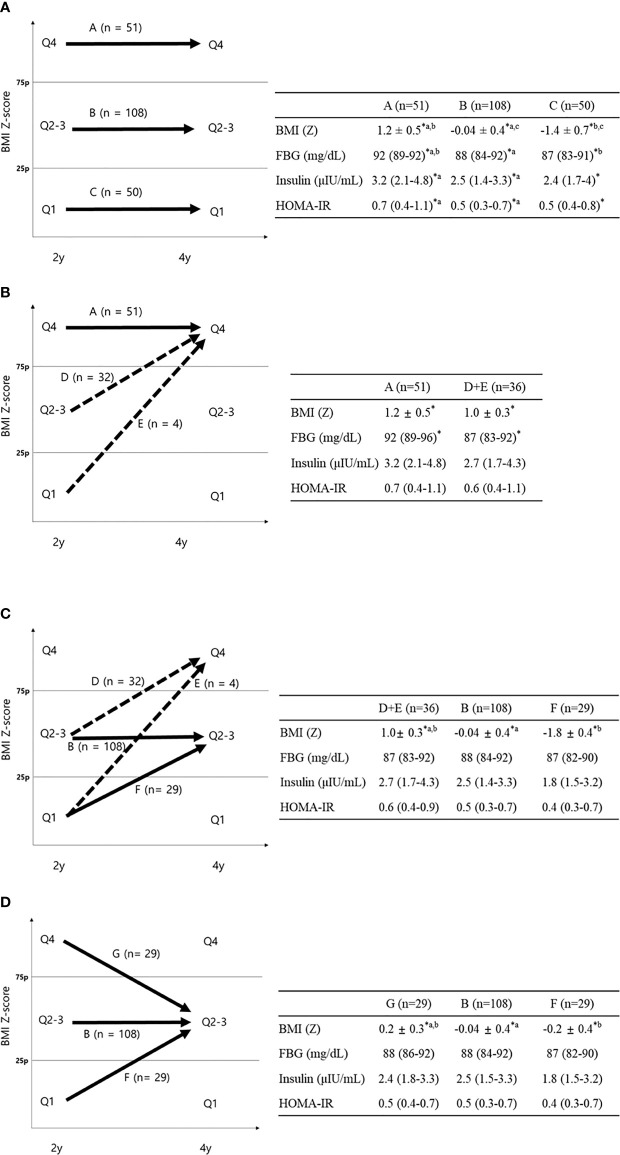
FBG, insulin, and HOMA-IR according to BMI Z-score quartile change. Data are shown as the mean ± standard deviation or median (interquartile range). *P < 0.05 between groups. **(A)** Comparison between groups **A–C**; ^a^ *P < *0.05 between groups **A** and **B**; ^b^ *P* < 0.05 between groups **A** and **C**; ^c^ *P* < 0.05 between groups B and C using Bonferroni *post-hoc* analysis. **(B)** Comparison between groups A and D+E (dotted line). **(C)** Comparison between groups **D+E**, **B**, and **F (D)** Comparison between groups **B**, **F**, and **G** ^a^p < 0.05 between groups **B** and **G**; ^b^ *P* < 0.05 between groups B and F using Bonferroni *post-hoc* analysis.

Statistical analyses were performed using SPSS ver. 26 for Windows software (SPSS Inc., Chicago, IL, USA). Variables were assessed for normality. Insulin and HOMA-IR were log-transformed due to their skewed distribution. Continuous variables were described as means ± standard deviations (SDs) or medians (interquartile ranges). Children were categorized into 3 groups according to the quartile of BMI (Z) at age 2 and age 4 as follows: Q4, ≥75th percentile; Q2–3, 25th to 75th percentile; and Q1, < 25th percentile. To compare the Q1, Q2–3, and Q4 groups at age 4, categorical variables were analyzed by the chi-squared test for trend analysis, and continuous variables were analyzed by the analysis of variance (ANOVA) with the Bonferroni *post-hoc* test. The mean BMI (Z) of ages 2 and 4 [(2-year BMI(Z) + 4-year BMI (Z))/2] and the change in BMI (Z) from age 2 to 4 [4-yr BMI (Z) – 2-yr BMI (Z)] were analyzed as continuous variables in the total population and within BMI (Z) quartiles at each age. Multiple regression analyses were performed after adjusting for age (months), sex, birth weight, gestational age, SGA, parental BMI, and maternal education. The variables that could plausibly influence the association and had an association with the outcomes at *P*<0.1 in univariate analysis were selected for adjustment (**Supplementary Table S1**). Then, changes in the BMI (Z) quartile from age 2 to 4 were tracked as shown in **Figures 1**, **2**. Children were classified into 7 change groups, labeled A to G, according to the change in BMI (Z) quartile from age 2 to 4. The children who were always in the highest, middle, or lowest BMI (Z) quartile (**Figure 2A**) were named groups A (Q4 → Q4, n = 51), B (Q2–3 → Q2–3, n = 108) and C (Q1 → Q1, n = 50), respectively. Children who went up in the BMI (Z) quartile from the age of 2 to 4 (**Figures 2B, C**) were called groups D (Q2-3 → Q4, n = 32), E (Q1 → Q4, n = 4), and F (Q1 → Q2-3, n = 29). The decreasing BMI (Z) quartile group was group G (Q4 → Q2-3, n = 29, **Figure 2D**). Glucose, insulin, and HOMA-IR levels were compared between these groups. Student’s t-test and ANOVA were run to evaluate differences in glucose, insulin, and HOMA-IR according to the change group. Linear regression analyses were conducted to evaluate whether the BMI (Z) change groups affected the level of glucose, insulin, and HOMA-IR, adjusted for age, sex, birth weight, gestational age, SGA, parental BMI, and maternal education level. A *P*-value < 0.05 was considered statistically significant.

### Ethics Statement

The present study protocol was reviewed and approved by the Institutional Review Board of Seoul National University Hospital (IRB No. 1201-010-392). Informed consent was provided by each subject’s parents. The protocol was in accordance with the Declaration of Helsinki and institutional guidelines.

## Results

### Characteristics of Total Subjects

The height, weight, and BMI were 86.3 ± 2.9 cm, 12.3 ± 1.4 kg, and 16.5 ± 1.5, respectively, at age 2 and 101.9 ± 3.7 cm, 16.4 ± 1.8 kg, and 15.7 ± 1.2 at age 4. BMI (Z) was -0.001 ± 1.0 at age 2 and -0.03 ± 1.0 at age 4. The gestational age was 38.6 ± 1.5 weeks, the birth weight was 3.2 ± 0.5 kg, and 23 children (6.9% of the sample) were born preterm. Of the infants, 115 (46.4%) were breastfed exclusively for ≥ 6 months. At the age-4 visit, FBG and fasting insulin were 89 (85–92) mg/dl and 2.5 (1.6–3.6) μIU/ml, respectively, and HOMA-IR was 0.6 (0.3–0.8). The maternal and paternal BMI at age 4 were 21.9 ± 3.0 and 25.0 ± 3.1, respectively. The mothers of 275 (82.3%) children had graduated from college (**Table 1**).

**Table 1:** Clinical characteristics of the participants.

	Total	Boys	Girls	*P*
No. of children	334	179 (53.7)	155 (46.3)	
At birth and infancy				
Gestation (weeks)	38.6 ± 1.5	38.6 ± 1.5	38.7 ± 1.4	0.542
Preterm birth	23 (6.9)	12 (6.7)	11 (7.1)	1.000
Birth weight (kg)	3.2 ± 0.5	3.2 ± 0.5	3.1 ± 0.5	0.029
Small for gestational age	17 (3.9)	7 (3.9)	10 (5.1)	0.326
Exclusive breastfeeding ≥ 6 months	155 (46.4)	77 (43.0)	78 (50.3)	0.189
Age 2				
Height (cm)	86.3 ± 2.9	86.8 ± 2.9	85.6 ± 2.8	<0.001
Height Z-score	0.01 ± 0.8	0.02 ± 0.8	0.004 ± 0.8	0.841
Weight (kg)	12.3 ± 1.4	12.7 ± 1.4	11.9 ± 1.3	<0.001
Weight Z-score	0.05 ± 1.0	0.08 ± 1.0	0.004 ± 0.9	0.464
BMI (kg/m^2^)	16.5 ± 1.5	16.8 ± 1.5	16.3 ± 1.4	0.001
BMI Z-score	-0.001 ± 1.0	0.2 ± 1.0	-0.2 ± 0.9	0.001
Age 4				
Height (cm)	101.9 ± 3.7	102.2 ± 3.7	101.5 ± 3.6	0.037
Height Z-score	0.2 ± 0.8	0.2 ± 0.8	0.2 ± 0.8	0.644
Weight (kg)	16.4 ± 1.8	16.6 ± 1.8	16.1 ± 1.8	0.024
Weight Z-score	0.1 ± 0.9	0.1 ± 0.9	0.2 ± 0.9	0.471
BMI (kg/m^2^)	15.7 ± 1.2	15.8 ± 1.2	15.6 ± 1.2	0.194
BMI Z-score	-0.03 ± 1.0	-0.04 ± 1.0	-0.02 ± 0.9	0.842
FBG* (mg/dl)	89 (85-92)	90 (86-94)	87 (83-91)	0.004
Fasting insulin* (μIU/ml)	2.5 (1.6-3.6)	2.4 (1.5-3.4)	2.7 (1.7-3.7)	0.135
HOMA-IR*	0.6 (0.3-0.8)	0.5 (0.3-0.8)	0.6 (0.4-0.8)	0.288
Maternal BMI (kg/m^2^)	21.9 ± 3.0	22.0 ± 3.2	21.8 ± 2.7	0.525
Paternal BMI (kg/m^2^)	25.0 ± 3.1	24.9 ± 3.2	25.0 ± 3.0	0.840
Maternal education ≥ college	275 (82.3)	140 (78.2)	135 (87.1)	0.043

Data are expressed as mean ± SD, median (interquartile range), or number (%).

*Log-transformed for statistical analysis.

BMI, body mass index; HOMA-IR, homeostatic model assessment of insulin resistance.

### Changes in BMI Z-score Quartile From Age 2 to 4

**Table 2** shows the characteristics of children according to the BMI (Z) quartile at age 4. The BMI (Z) of Q1, Q2–3, and Q4 at age 4 was -1.3 ± 0.6, -0.02 ± 0.4, and 1.1 ± 0.4, respectively (*P* < 0.001). BMI (Z) at age 2 was -0.8 ± 0.7, 0.02 ± 0.8, and 0.7 ± 0.9 in the children who were in Q1, Q2–3, and Q4 at age 4, respectively (*P*<0.001). A total of 0%, 9%, and 35.6% of the Q1, Q2–3, and Q4 groups at age 4 were overweight or obese at age 2, respectively (*P* for trend <0.001). When comparing the 4-year-old BMI (Z) quartiles, FBG was higher in Q4 than in Q2–3 and Q1 (90 vs. 88 and 87 mg/dl, *P* < 0.05 for both), and insulin and HOMA-IR were higher in Q4 than in Q2–3 at age 4 (3.0 vs. 2.3 μIU/ml, *P* = 0.009 for insulin; 0.7 vs. 0.5, *P* = 0.004 for HOMA-IR).

**Table 2:** Comparison of characteristics between BMI Z-score quartiles at age 4.

	BMI quartile at age 4			
	Lowest quartile (Q1)	Middle quartiles (Q2–3)	Highest quartile (Q4)	*P*
No. of children	81 (24.3)	166 (49.7)	87 (26.0)	
Boys	37 (45.7)	91 (54.8)	51 (58.6)	0.096
At birth and infancy				
Gestation (weeks)	38.6 ± 1.3	38.5 ± 1.5	38.7 ± 1.5	0.609
Preterm birth, n (%)	3 (3.7)	14 (8.4)	6 (6.9)	0.431
Birth weight (kg)	3.1 ± 0.5	3.2 ± 0.5	3.3 ± 0.5^b^	0.007
SGA	6 (7.4)	9 (5.4)	2 (2.3)	0.131
Exclusive breastfeeding ≥ 6 months	35 (43.2)	85 (51.2)	35 (40.2)	0.667
Age 2				
BMI Z-score	-0.8 ± 0.7	0.02 ± 0.8^a^	0.7 ± 0.9^b,c^	<0.001
Overweight or obese at age 2	0 (0.0)	15 (9.0)	31 (35.6)	<0.001
BMI quartile group (Q1/Q2–3/Q4)	50/30/4 (61.7/37.0/1.2)	29/108/29 (17.5/65.1/17.5)	4/32/51 (4.6/36.8/58.6)	<0.001
Age 4				
BMI Z-score	-1.3 ± 0.6	-0.02 ± 0.4^a^	1.1 ± 0.4^b,c^	<0.001
FBG* (mg/dl)	87 (85-91)	88 (84-91)	91 (86-94)^b,c^	0.002
Fasting insulin* (μIU/ml)	2.3 (1.6-3.5)	2.4 (1.5-3.2)	3.0 (1.9-4.5)^b^	0.011
HOMA-IR*	0.5 (0.3-0.8)	0.5 (0.3-0.7)	0.7 (0.4-1.0)^b^	0.005
Maternal BMI (kg/m^2^)	21.2 ± 2.5	21.8 ± 3.3	22.6 ± 3.0^c^	0.009
Paternal BMI (kg/m^2^)	24.0 ± 2.6	25.6 ± 3.2^a^	25.0 ± 3.1^c^	0.005
Maternal education ≥ college	67 (82.7)	135 (81.3)	73 (83.9)	0.830

Data are expressed as mean ± SD, median (interquartile range), or number (%).

P-value, analysis of variance (ANOVA) for continuous variables, and chi-squared test for trend analysis for categorical variables.

^a^P <0.05 between Q1 and Q2–3; ^b^P <0.05 between Q1 and Q4; ^c^P <0.05 between Q2–3 and Q4 using Bonferroni post-hoc analysis.

BMI, body mass index; HOMA-IR, homeostatic model assessment of insulin resistance; SGA, small for gestational age.

*Log-transformed for statistical analysis.

**Figure 3:**
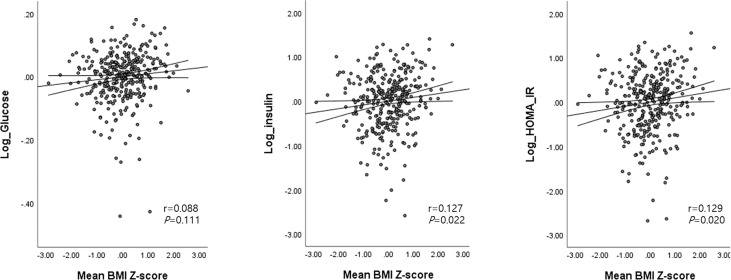
Associations between the mean BMI Z-score at ages 2 and 4 with glucose, insulin, and HOMA-IR levels at age 4. Age (months), sex, birth weight, gestational age, SGA, parental BMI, and maternal education were adjusted as covariates. Mean BMI Z-score at ages 2 and 4 = [(BMI Z-score at age 2 + BMI Z-score at age 4)/2].

The mean BMI (Z) at ages 2 and 4 was correlated with higher FBG (β=0.2, 95% confidence interval [CI]: 0.005, 0.03, *P* = 0.004), insulin (β=0.1, 95% CI: 0.04, 0.20, *P* = 0.005), and HOMA-IR (β = 0.2, 95% CI: 0.05, 0.22, *P* = 0.003). Insulin and HOMA-IR were still positively associated with mean BMI (Z) after adjustment for age, sex, gestational age, birth weight, SGA, parental BMI, and maternal education (*P* = 0.022 for insulin and *P* = 0.02 for HOMA-IR) (**Figure 3**).

The BMI (Z) change between ages 2 and 4 was not associated with FBG, insulin, or HOMA-IR levels in the analysis of the total population. When subgroup analyses were performed according to the BMI (Z) quartile at each age, the inverse relationship between BMI (Z) change [4-year BMI (Z) – 2-year BMI (Z)] and FBG was only significant in the Q4 group at age 4. In detail, the higher the BMI (Z) at age 2 compared to the BMI (Z) at age 4 [a persistent high BMI (Z) at both ages 2 and 4], the higher the FBG at age 4 (β = -0.3, 95% CI: -0.05, -0.005, *P*=0.013) among 4-year-old children in Q4, after adjusting for age, sex, birth weight, gestational age, SGA, parental BMI, and maternal education (**Supplementary Figure S2**). There was no correlation between the BMI (Z) change and insulin or HOMA-IR in each BMI (Z) quartile subgroup at ages 2 and 4.

Changes in the BMI (Z) quartile between ages 2 and 4 are described in **Figure 1** (*P* for trend < 0.001). A total of 190 children (56.9% of the total) remained in the same BMI (Z) quartile from age 2 to 4 [Q1→Q1, n=50 (61.7%); Q2–3→Q2–3, n=108 (65.1%); Q4 →Q4, n=51 (58.6%)]. A total of 65 children (19.4% of the total) moved to a higher BMI (Z) quartile from age 2 to age 4 (Q1→Q2–3, n=29; Q1→Q4, n=4; Q2-3→Q4, n=32). A total of 58 children (17.7% of total) moved to a lower BMI (Z) quartile from age 2 to age 4 (Q2-3→Q1, n=30; Q4→Q2–3, n=29; Q4→Q1, n=1).

### Fasting Blood Glucose, Insulin, and Homeostatic Model Assessment of Insulin Resistance According to Change in BMI Z-Score Quartile From Age 2 to 4

The FBG, insulin, and HOMA-IR at age 4 were significantly different between group A (Q4→4), group B (Q2-3→2-3), and group C (Q1→1) (92 vs. 88 vs. 87 mg/dl, *P <*0.001 for FBG; 3.2 vs. 2.5 vs. 2.4 μIU/ml, *P* = 0.008 for insulin; 0.7 vs. 0.5 vs. 0.5, *P* = 0.003 for HOMA-IR, **Figure 2A**). As shown by Bonferroni *post-hoc* analysis, FBG, insulin, and HOMA-IR were higher in group A than in group B (*P* < 0.001 for FBG, *P* = 0.003 for insulin, and *P* = 0.001 for HOMA-IR) and group C (*P* = 0.002 for FBG), without differences between groups B and C. Multiple regression analysis showed significantly higher levels of glucose, insulin, and HOMA-IR in group A than in group B and higher HOMA-IR in group A than in group C (*P <*0.05 between two groups for all variables, **Table 3**).

**Table 3:** Regression analysis of FBG, insulin, and HOMA-IR according to BMI Z-score change group.

	Analysis 1 [Figure 2A]			Analysis 2 [Figure 2B]			Analysis 3 [Figure 2C]			Analysis 4 [Figure 2D]		
Group	FBG	Insulin	HOMA	FBG	Insulin	HOMA	FBG	Insulin	HOMA	FBG	Insulin	HOMA
A (Q4→4)	ref	ref	ref	ref	ref	ref	–	–	–	–	–	–
B (Q2–3→2–3)	-0.3 (0.01)^**^	-0.3 (0.1)^*^	-0.3 (0.1)^**^	–	–	–	ref	ref	ref	ref	ref	ref
C (Q1→1)	-0.2 (0.02)	-0.2 (0.1)	-0.2 (0.1)^*^	–	–	–	–	–	–	–	–	–
D+E (Q1–3→4)	–	–	–	-0.3 (0.02) ^**^	-0.2 (0.2)	-0.2 (0.2)	-0.003 (0.02)	0.06 (0.1)	0.06 (0.1)	–	–	–
F (Q1→2–3)	–	–	–	–	–	–	-0.09 (0.02)	-0.02 (0.1)	-0.03 (0.2)	-0.08 (0.02)	-0.03 (0.1)	-0.04 (0.1)
G (Q4→2–3)	–		–	–	–	–	–	–	–	-0.02 (0.02)	0.1 (0.1)	0.1 (0.2)

Values are shown as β (standard error). Glucose, insulin, and HOMA-IR were log-transformed for analysis.

Sex, age (months), birth weight, gestational age, small for gestational age, parental BMI, and maternal education were adjusted for all analyses.

FBG, fasting blood glucose; HOMA-IR, homeostatic model assessment of insulin resistance; ref, reference, *P < 0.05, **P < 0.01.

Looking only at the 4-year-old children in the highest BMI (Z) quartile (Q4), we compared the continuously highest (group A, Q4→4) with the increasing BMI (Z) groups (group D+E, Q1-3→4, dotted arrow in **Figure 2B**). Group A showed significantly higher FBG than group D+E (92.2 vs. 87.3 mg/dl, *P <*0.001, **Figure 2B**), without differences in fasting insulin or HOMA-IR. The FBG difference between group A and group D+E was also significant after adjusting for covariates (*P*=0.004, **Table 3**).

No differences in FBG, insulin, or HOMA-IR were found between the two increasing BMI (Z) quartile groups (group D+E, Q1–3→4 and group F, Q1→2–3) and group B (Q2–3→2-3, **Figure 2C**) despite different BMIs at age 4. Among 4-year-old children in the middle BMI (Z) quartile (Q2–3), we compared them according to their 2-year-old BMI (Z) (group G, Q1→2–3; group B, Q2-3→2–3; and group F, Q4→2–3, solid arrows in **Figure 2D**). There were no significant differences in FBG, insulin, or HOMA-IR between the three groups, suggesting that the 4-year IR within the middle BMI (Z) group (Q2–3) was determined by the 4-year-old BMI (Z) quartile regardless of the 2-year BMI (Z) quartile (**Figure 2D**).

## Discussion

Being in the highest BMI (Z) quartile at both ages 2 and 4 was associated with higher FBG and HOMA-IR at age 4 than being persistently in the lowest or middle BMI (Z) quartile or increasing in the BMI (Z) quartile. When we analyzed only the middle BMI (Z) quartile at age 4, FBG and HOMA-IR were similar irrespective of the previous BMI (Z) quartile at age 2. When we excluded the persistently highest BMI (Z) quartile group, FBG and HOMA-IR were not different between the children in the highest and middle BMI quartiles at age 4.

The differences in FBG and HOMA-IR were significant between the children who remained in the highest, middle, and lowest BMI (Z) quartiles at ages 2 and 4 in this study, even though these children were only 4 years old. There have been few pediatric reports on FBG or IR in preschool-age children (17–19), and most have focused on SGA (17, 18). The children born SGA have had higher fasting insulin at age 3 than those born appropriate for gestational age (AGA) (17). One pediatric study reported an inverse relationship between BMI and HOMA-IR in 5- and 6-year-old children in both sexes, although this analysis was limited by its cross-sectional nature (18). The current longitudinal study serially assessed young children at ages 2 and 4, most of whom were born AGA, demonstrating the adverse effect of persistently high BMI (Z) quartile rather than increasing BMI (Z) quartile on FBG and HOMA-IR at age 4.

This study was strengthened by not only considering BMI at age 4 but also tracking BMI between ages 2 and 4 to evaluate its effect on FBG and HOMA-IR at age 4. A continuously high level of adiposity in early childhood adversely affected glucose homeostasis. Although this study evaluated FBG and HOMA-IR in 4-year-old children, our results are consistent with previous findings (20–22), supporting the close association between the duration of obesity and impaired glucose metabolism in adults. Although it was in the normal range, HOMA-IR was significantly higher in children within the highest BMI group at both ages 2 and 4 in this study. This finding indicates that the difference in IR according to BMI starts in early childhood, at as young as 4 years of age. Whether early childhood adiposity and its duration may affect glucose homeostasis after puberty needs to be investigated.

The BMI (Z)-adjusted difference in FBG at age 4 between group A (Q4→4, persistently high adiposity) and group D+E (Q1-3→4, increasing adiposity) suggested the potential adverse impact of a long-term high adiposity on later glucose metabolism. This impact was supported by not only the positive relationship between the mean BMI (Z) between ages 2 and 4 and FBG at age 4 but also the higher FBG among the 4-year-old highest BMI (Z) quartile with small changes in BMI between ages 2 and 4 (a persistently high BMI from age 2 to 4). Previous longitudinal studies showing higher metabolic risks after age 6 in children with increasing BMI (Z) from 9 months to 6 years of age (23), a strong relationship of BMI increase between 2 and 6 years with insulin level at age 6 (17), and higher HOMA-IR at age 12 in children whose BMI increased from age 1.5 to 3 years (24) support the adverse metabolic effect of increasing BMI during childhood. Although neither glucose nor insulin at age 4 differed between groups D+E (Q1-3→4), B (Q2-3→2-3) and F (Q1→2-3) in our study, their effect on later obesity and metabolic risk during childhood and adolescence needs to be evaluated.

The three Q2–3 groups at age 4 (groups B, F, and G) showed similar glucose and insulin levels irrespective of the BMI quartile at age 2. Going from overweight or obesity to normal weight may reverse or restore glucose metabolism in young children. Despite the age difference, our findings are in line with a Danish cohort study showing that the individuals who were overweight at age 7 but returned to normal weight by age 13 and maintained a normal weight until young adulthood showed a similar risk of T2DM in their 30s to 60s as those who maintained a normal weight throughout life (25).

This study sample was made up of a prospective birth cohort that we followed to age 4. All included subjects were healthy young children, and most of them were born AGA. Our study is limited by its short follow-up period and the use of noninvasive HOMA-IR for assessing IR but is strengthened by the evaluation of young-childhood IR. Further longitudinal evaluation of this prospective cohort is anticipated.

## Conclusion

The children who stayed in the highest BMI (Z) quartile from age 2 to 4 had higher FBG and IR at age 4 than any other BMI (Z) change group. Further longitudinal studies are warranted to determine the effects of early-life changes in BMI and IR on later obesity and glucose metabolism.

## Data Availability Statement

The original contributions presented in the study are included in the article/**Supplementary Material**. Further inquiries can be directed to the corresponding author.

## Ethics Statement

The studies involving human participants were reviewed and approved by the Institutional Review Board of Seoul National University Hospital (IRB No. 1201-010-392). Written informed consent to participate in this study was provided by the participants’ legal guardian/next of kin.

## Author Contributions

HJL conceptualized the study, collected data, carried out the analyses, and drafted the manuscript. YHL designed the data collection of the cohort and critically reviewed the manuscript. YCH established the cohort, acquired funding for the study, and critically reviewed the manuscript. CHS coordinated and supervised data collection and critically reviewed the manuscript for important intellectual content. YAL conceptualized the study, coordinated and supervised data collection, and critically reviewed and revised the manuscript for important intellectual content. All authors approved the final manuscript as submitted and agree to be accountable for all aspects of the work.

## Funding

This research was funded by the Ministry of Environment through the Environmental Health Center Program of the Republic of Korea and a grant (18162MFDS121) from the Ministry of Food and Drug Safety in 2018.

## Conflict of Interest

All the authors declare that the research was conducted in the absence of any commercial or financial relationships that could be construed as a potential conflict of interest.

## Publisher’s Note

All claims expressed in this article are solely those of the authors and do not necessarily represent those of their affiliated organizations, or those of the publisher, the editors and the reviewers. Any product that may be evaluated in this article, or claim that may be made by its manufacturer, is not guaranteed or endorsed by the publisher.
