# Supplementary material for: Body Mass Index Changes and Insulin Resistance at Age 4: A Prospective Cohort Study
Source: Front Endocrinol (Lausanne). 2022 May 23;13:872591. doi: 10.3389/fendo.2022.872591 (PMC9169890; doi:10.3389/fendo.2022.872591)
Supplement: Supplementary file 1 [file DataSheet_1.docx]

Supplementary Material

# Supplementary Tables

**Supplementary Table S1.** Simple regression analysis of each exposure with fasting blood glucose, insulin and HOMA-IR

|  | Fasting blood glucose | | Insulin | | HOMA-IR | |
| --- | --- | --- | --- | --- | --- | --- |
|  | B (S.E.) | *P* | B (S.E.) | *P* | B (S.E.) | *P* |
| Age (months) | 0.003 (0.003) | 0.318 | 0.047 (0.023) | 0.045 | 0.050 (0.025) | 0.046 |
| Girls | -0.026 (0.009) | 0.004 | 0.118 (0.069) | 0.135 | 0.081 (0.076) | 0.288 |
| Breastfeeding | 0.00 (0.009) | 0.978 | 0.056 (0.071) | 0.429 | 0.057 (0.076) | 0.456 |
| Birth weight | 0.034 (0.009) | <0.001 | 0.019 (0.073) | 0.790 | 0.053 (0.078) | 0.496 |
| SGA | -0.04 (0.02) | 0.047 | 0.048 (0.162) | 0.769 | 0.007 (0.172) | 0.967 |
| Weeks of gestation | 0.005 (0.003) | 0.079 | 0.030 (0.024) | 0.212 | 0.036 (0.026) | 0.168 |
| Preterm birth | -0.003 (0.018) | 0.884 | 0.166 (0.140) | 0.238 | 0.163 (0.149) | 0.276 |
| Paternal BMI | 0.003 (0.001) | 0.082 | 0.035 (0.011) | 0.002 | 0.038 (0.012) | 0.002 |
| Maternal BMI | 0.004 (0.001) | 0.004 | 0.016 (0.012) | 0.177 | 0.020 (0.013) | 0.108 |
| Maternal education | -0.024 (0.012) | 0.039 | -0.023 (0.093) | 0.809 | -0.047 (0.099) | 0.638 |

BMI, body mass index; Breastfeeding, Exclusive breastfeeding ≥ 6 months; HOMA-IR, Homeostatic model assessment of insulin resistance; Maternal education, Maternal education ≥ college ; SGA, small for gestational age

**Supplementary Table S2.** Characteristics of according to birth history

|  | Term | | | Preterm | | |
| --- | --- | --- | --- | --- | --- | --- |
|  | Total term | Term AGA | Term SGA | Total preterm | Preterm AGA | Preterm SGA |
| N | 311 | 298 | 13 | 23 | 19 | 4 |
| 2yr Height Z-score | 0.06 (-0.5, 0.6) | 0.08 (-0.5, 0.08) | -0.5 (-0.9, -0.1)* | -0.4 (-0.7, 0.6) | -0.1 (-0.6, 0.6) | -0.7 (-1.2, -0.6) |
| 4yr Height Z-score | 0.3 (-0.4, 0.8) | 0.3 (-0.3, 0.8) | -0.2 (-0.7, 0.2)* | 0.1 (-0.4, 0.6) | 0.3 (-0.4, 0.6) | -0.4 (-0.7, 0.001) |
| 2yr Weight Z-score | -0.01 (-0.6, 0.7) | 0.01 (-0.6, 0.7) | -0.4 (-1.6, 0.06)* | 0.2 (-0.9, 0.6) | 0.1 (-0.9, 0.3) | 0.6 (-0.8, 0.8) |
| 4yr Weight Z-score | 0.2 (-0.6, 0.8) | 0.2 (-0.5, 0.8) | -0.7 (-1.1, -0.2)* | 0.1 (-0.5, 0.8) | 0.1 (-0.5, 0.8) | -0.01 (-0.9, 0.6) |
| 2yr BMI Z-score | -0.1 (-0.5, 0.6) | -0.1 (-0.5, -0.1) | -0.02 (-1.2, 0.3) | -0.3 (-0.6, 1.4) | -0.5 (-0.7, 0.8) | 1.2 (0.1, 1.7) |
| 4yr BMI Z-score | 0.002 (-0.7, 0.6) | 0.04 (-0.6, 0.6) | -0.4 (-1.6, -0.1)* | 0.05 (-0.4, 0.8) | 0.1 (-0.4, 0.6) | 0.4 (-0.4, 1.0) |
| Glucose | 89 (84, 92) | 89 (85, 92) | 86 (83, 88)* | 87 (85, 94) | 90 (85, 94) | 84 (69, 95) |
| Insulin | 2.5 (1.6, 4,2) | 2.5 (1.6, 3.5) | 2 (1.5, 3.9) | 2.8 (1.6, 3.6) | 2.8 (1.6, 4.2) | 3.0 (1.7, 6.0) |
| HOMA-IR | 0.5 (0.3, 0.8) | 0.6 (0.3, 0.8) | 0.4 (0.3, 0.8) | 0.6 (0.3, 1.0) | 0.6 (0.3, 1.0) | 0.6 (0.3, 1.4) |

Data are shown as median (interquartile range)

*P<0.05 for Mann–Whitney U test between term AGA and term SGA

There were no significant differences between term and preterm birth or between preterm AGA and SGA

AGA, appropriate for gestational age; HOMA-IR, Homeostatic model assessment of insulin resistance; SGA, small for gestational age

# Supplementary Figures


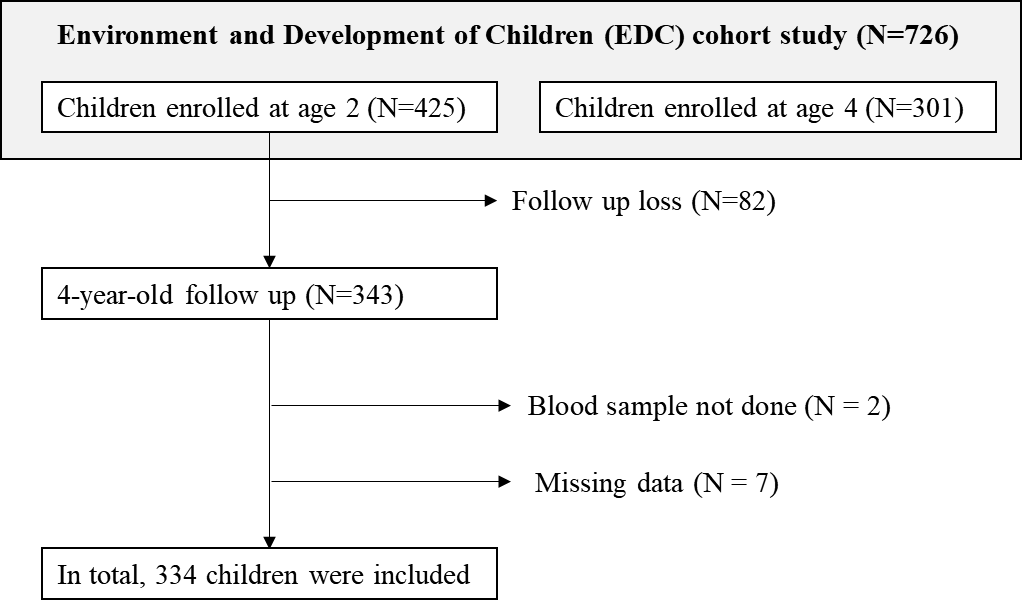


**Supplementary Figure S1.** Study participants of the Environment and Development of Children (EDC) cohort study.

~~.
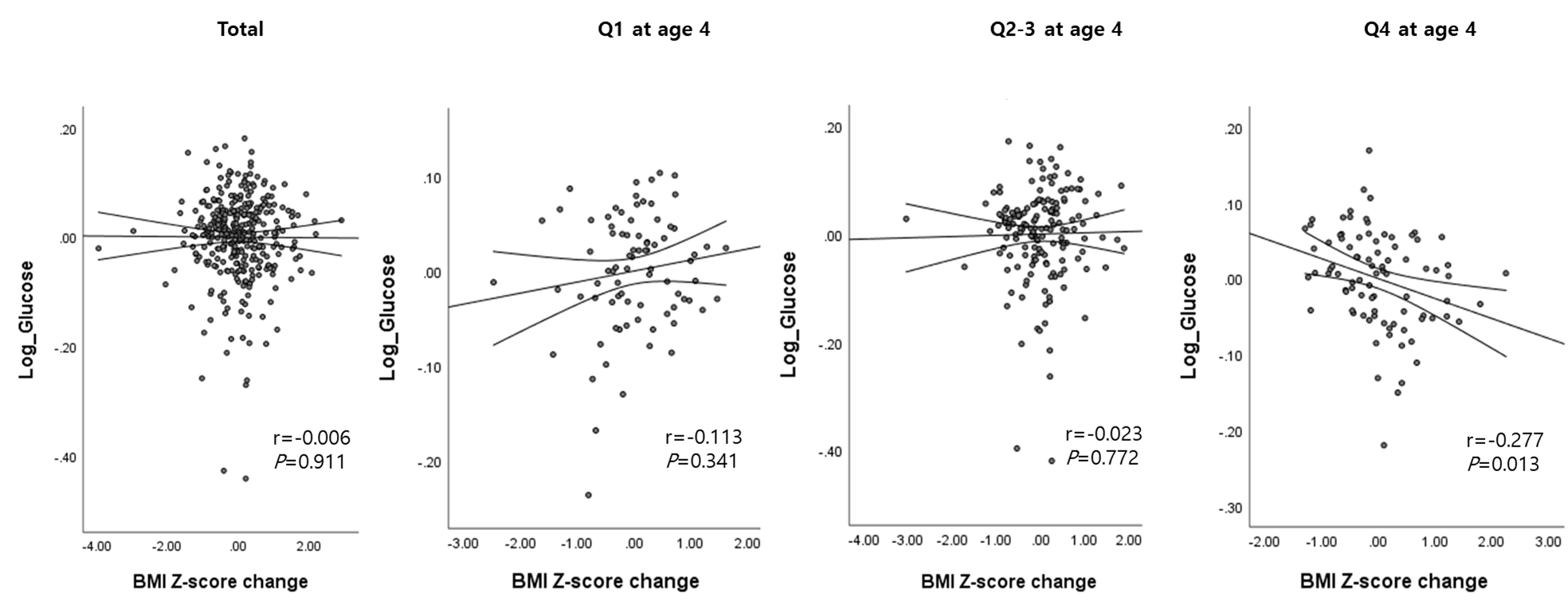
~~

**Supplementary Figure S2**. Correlation between the BMI Z-score change and glucose in the total population and among each BMI Z-score quartile group (Q1, Q2-3, and Q4) at age 4.

The funding information and grant number is correctly included

The funding information and grant number are correctly included
